# Global changes in *Staphylococcus aureus* virulence and metabolism during colonization of healthy skin

**DOI:** 10.1128/iai.00028-25

**Published:** 2025-03-21

**Authors:** Timothy J. Enroth, Morgan M. Severn, Flavia G. Costa, Alyson R. Bovee, Reid V. Wilkening, Dustin T. Nguyen, Christophe Langouët-Astrié, Alexander R. Horswill

**Affiliations:** 1 Department of Immunology and Microbiology, University of Colorado School of Medicine 12225, Aurora, Colorado, USA; 2 Department of Dermatology, Duke University 3065, Durham, North Carolina, USA; 3 Section of Pediatric Critical Care Medicine, Department of Pediatrics, University of Colorado School of Medicine 12225, Aurora, Colorado, USA; 4 Division of Pulmonary Sciences and Critical Care, University of Colorado Denver 12226, Denver, Colorado, USA; 5 Department of Veterans Affairs Eastern, Colorado Healthcare System 20677 , Grand Junction, Colorado, USA; St Jude Children's Research Hospital, Memphis, Tennessee, USA

**Keywords:** transcriptomics, host-pathogen interactions, RNA sequencing, skin microbiota, *Staphylococcus aureus*, antibiotic resistance, bacteriology

## Abstract

*Staphylococcus aureus* and its antibiotic-resistant derivative, methicillin-resistant *S. aureus* (MRSA), are the leading causative agents of skin and soft tissue infections globally. *S. aureus* transiently colonizes the skin of healthy adults, and this transient colonization likely precedes an active infection. In recent years, there have been efforts to elucidate specific factors that help MRSA transition to an active infection, but the specific genetic determinants required for this transition following skin colonization are largely unknown. To address this question, we developed a model of asymptomatic colonization of mouse skin by MRSA. From this model, we could determine the MRSA and mouse transcriptional profiles by RNA sequencing (RNAseq) at 5- and 24-hour post-colonization. The *fadXDEBA* locus, required for fatty acid metabolism, was highly upregulated in our data, as were numerous virulence factors. RNAseq data were confirmed via functional *in vitro* and *in vivo* promoter-fusion assays using live bioluminescent imaging of the *fadXDEBA* locus promoter driving *fadB* transcription. We analyzed the functional capacity of members of the *fadXDEBA* locus, which encode crucial enzymatic components of the *S. aureus* β-oxidation pathway. The genes *fadD* and *fadA* modulate MRSA resistance to fosfomycin and other oxidative stressors during growth in the presence of the common skin fatty acid, palmitic acid. Overall, our data demonstrate that there are global changes to the MRSA transcriptome, priming the bacteria for survival by upregulation of known virulence factors and metabolic genes implicated in host skin-nutrient utilization.

**IMPORTANCE:**

*Staphylococcus aureus* is a major global agent of skin and soft tissue infections. *S. aureus* colonizes the skin transiently, an important precursor to infection. However, little is known about how *S. aureus* adapts to the skin at the transcriptional level. This study provides an overview of the *S. aureus* transcriptome during mouse skin colonization via RNA sequencing. We identified that the most highly upregulated genes during colonization are related to fatty acid metabolism. The disruption of certain genes in the fatty acid degradation pathway altered resistance of *S. aureus* to the antibiotic fosfomycin. This study provides an important step in understanding the transcriptional changes that occur during *S. aureus* skin colonization and may reveal novel targets of therapeutic interest for preventing skin infections.

## INTRODUCTION

*Staphylococcus aureus* is a highly successful and adaptable pathogen that colonizes a large percentage of the healthy adult population (1, 2). Possessing a vast arsenal of virulence factors including toxins, adhesins, and other surface-anchored proteins, *S. aureus* can infect and colonize a variety of niches. The most common infections are superficial skin and soft tissue infections (SSTI) (3); however, *S. aureus* can also cause severe invasive disease such as sepsis and osteomyelitis (4, 5). Treatment of such infections is complicated by the formation of persistent biofilms during chronic infections, as well as the widespread and global circulation of antibiotic-resistant derivatives of the organism*,* especially the methicillin-resistant *S. aureus* (MRSA) strains (6–12).

While *S. aureus* is an important pathogen, it has the capacity to asymptomatically colonize healthy humans. Approximately 20%–30% of all humans are colonized by *S. aureus* at any given time, primarily within the anterior nares (13). Though the primary reservoir is within the anterior nares, transient skin colonization does occur at a level typically less than 5% for sampled individuals (14, 15). Though transient, this colonization of the skin is thought to be important in the development of SSTIs, especially if the skin barrier is compromised such as in patients with atopic dermatitis (16). Colonization by *S. aureus* has also been shown to directly contribute to a significantly higher probability of invasive infection in general, further highlighting how colonization of the anterior nares is an important precursor to infection (17, 18). Despite the critical role of colonization of the skin, a complete understanding of how *S. aureus* adapts to the intrinsically antimicrobial and nutrient-limiting skin environment remains unclear.

To help address the unknowns of how *S. aureus* colonizes the skin and what factors may be important, we leveraged a new RNA sequencing (RNAseq) model to determine the differential expression of *S. aureus* and host genes following inoculation onto intact and undamaged murine skin. Our transcriptional profiling revealed that members of the *fadXEDBA* locus are the most highly differentially expressed genes when MRSA is introduced onto the lipid-rich healthy skin environment. Importantly, the skin is known to contain a variety of fatty acids, many of which are antimicrobial in nature but may also serve as an important nutrient source (19). Recently, the *fadXDEBA* locus was found to be important for the metabolic utilization of the skin-relevant fatty acid, palmitic acid, likely via conventional β-oxidation (20). This highlights how MRSA may be actively utilizing skin fatty acids during colonization.

MRSA-lipid interactions at the skin interface remain sparsely characterized, so we aimed at building on previous literature to begin assessing how skin lipids might broadly affect MRSA physiology during skin colonization. To build on our skin colonization RNAseq data, we sought to confirm our results using promoter-fusion luminescent reporters to analyze promoter responses to skin-relevant conditions both *in vivo* and *in vitro*. Finally, we assessed the contribution of various genes within the *fadXEDBA* locus to resistance against the antibiotic fosfomycin and the oxidative stressors sodium hypochlorite (NaOCl) and paraquat. Together, our data reveal vast MRSA transcriptional changes that occur during skin colonization, and we demonstrate that our skin colonization model and RNAseq data set can be used as starting points to effectively study MRSA physiology during the colonization of healthy skin.

## RESULTS

### Healthy murine skin colonization RNAseq

**Fig 1:**
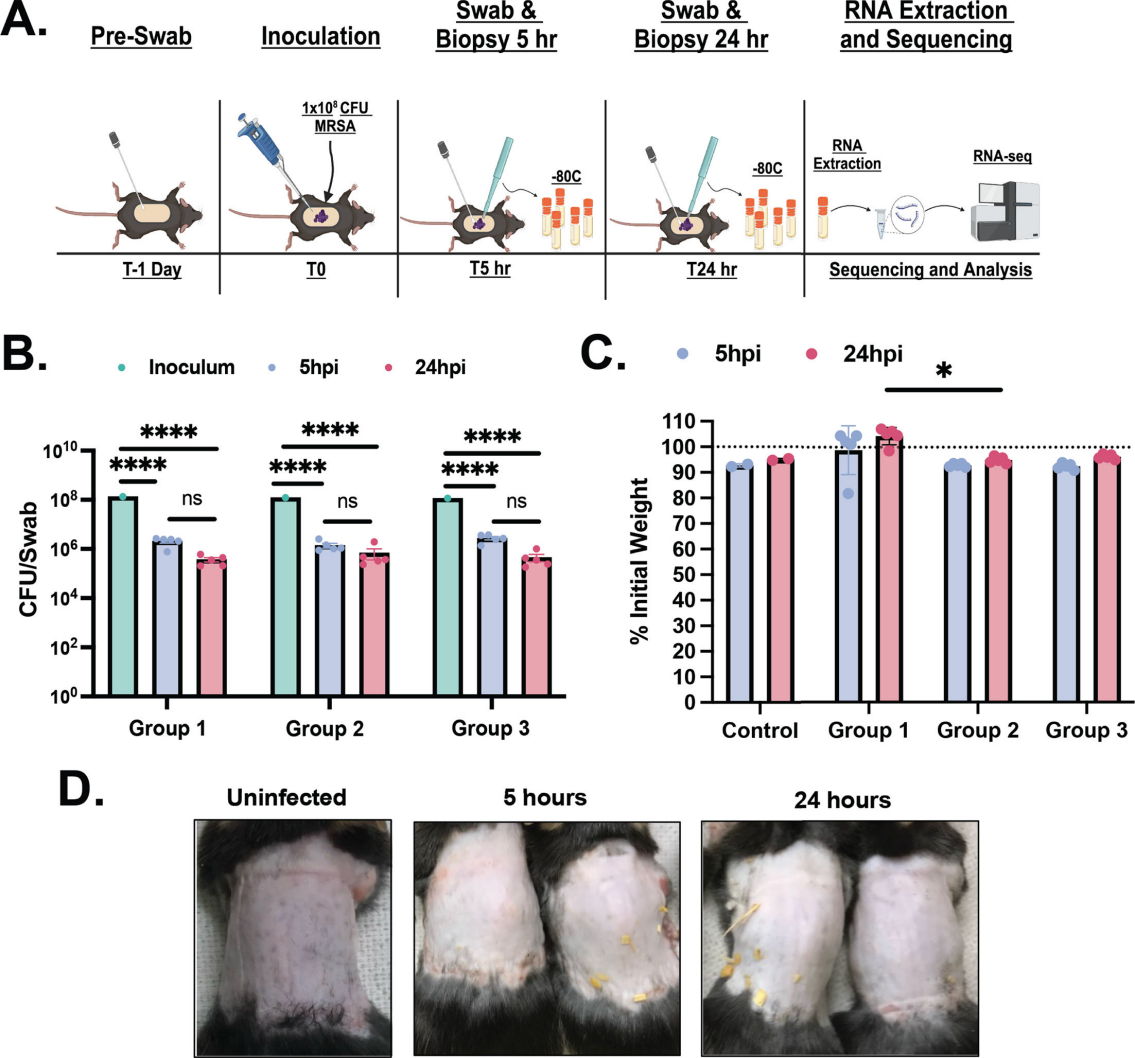
RNAsequencing experimental design. (A) Experimental scheme for the RNAseq experiment. (B) CFU/swab changes over time for mice colonized with MRSA. (C) Weight changes for colonized mice per group over time. (D) Representative images of mice backs colonized with MRSA at 5- and 24-hour post-inoculation (HPI) show no inflammation or signs of infection compared to the uncolonized control. * Indicates *P* ≦ 0.05, and **** indicates *P* ≦ 0.0001 by an ordinary one-way analysis of variance (ANOVA).

**Fig 2:**
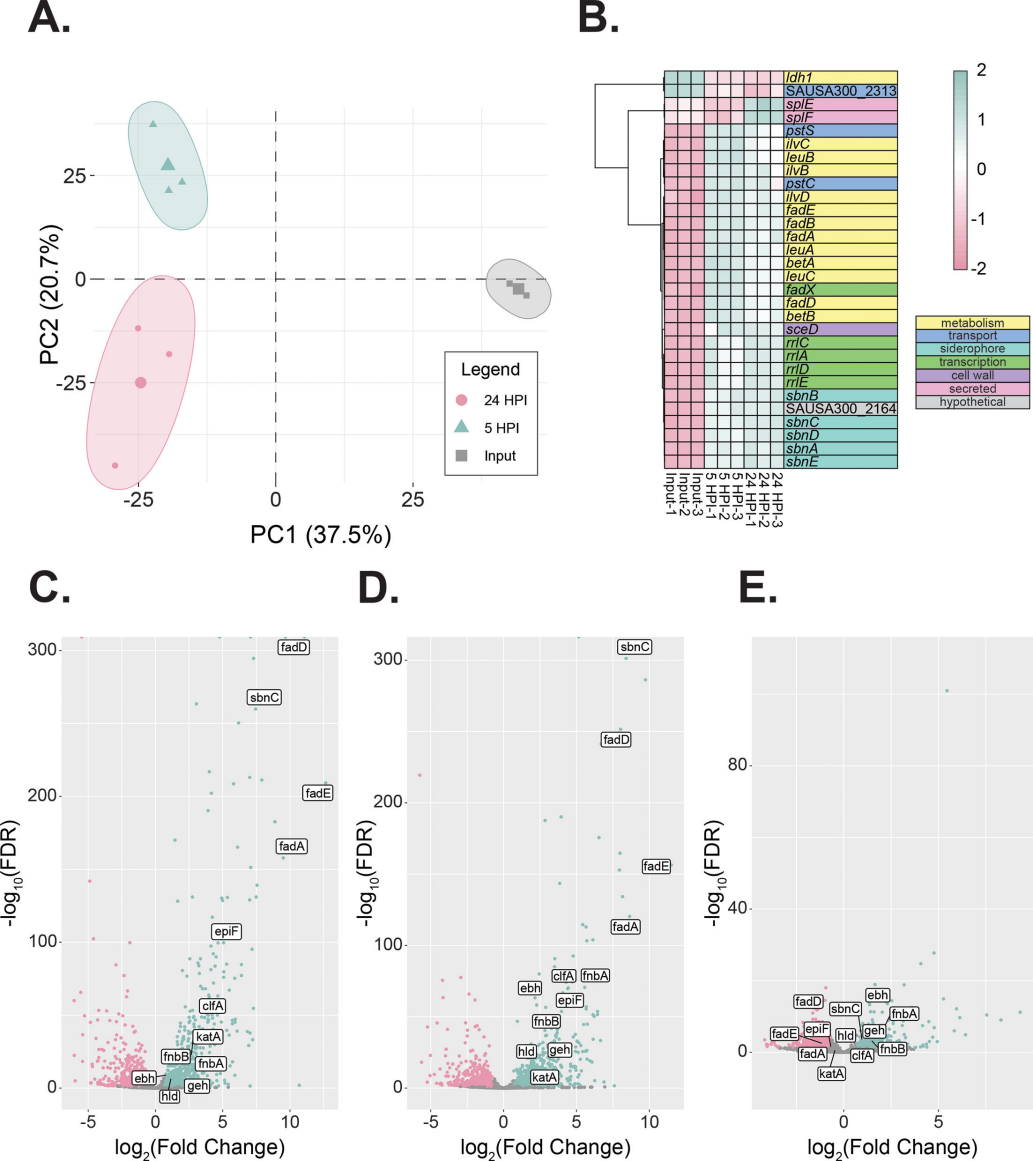
MRSA transcriptomic changes by RNAseq. (A) PCA of MRSA RNAseq for the input, 5- and 24-hour post-inoculation samples. (B) Heatmap of the top differentially expressed MRSA genes and their predicted functions, showing relative variance for each gene across each condition tested. Volcano plots for the most differentially expressed genes at 5 hours (C), 24 hours (D), and a comparison between 24 HPI and 5 HPI (E).

To better understand how *S. aureus* responds to skin colonization at the transcriptional level, we developed a model of intact murine skin colonization using AH1263 (hereafter referred to as MRSA), which is a plasmid-cured derivative of the USA300 MRSA strain LAC (Fig. 1A) (21). In this experiment, 10^8^ colony-forming units (CFUs) of MRSA were inoculated to the back skin of healthy C57BL/6J mice 24-hour post-depilation. At 5- and 24-hour post-inoculation (HPI), the colonized regions were swabbed for MRSA CFU enumeration, and mouse tissue was obtained post-sacrifice to determine the transcriptional response to colonization via dual RNAseq. Dual RNAseq is a method that can be used to simultaneously gather transcriptomic information from the host and bacteria from the same samples (22). We observed no difference in CFU enumeration between groups at 5 and 24 hours (Fig. 1B). Furthermore, MRSA-colonized mice showed no significant weight loss compared to control mice (Fig. 1C). The colonized skin had no visual signs of infection and grossly appeared the same as the uncolonized controls (Fig. 1D). We chose to analyze the MRSA transcriptional changes at 5- and 24-HPI, which we reasoned would allow us to analyze the MRSA transcriptome in response to early skin colonization and to account for the limited timeframe that this pathogen colonizes the skin of C57BL/6J mice. When analyzing the bacterial transcripts, principal component analysis (PCA) shows the separation of both the 5 and 24 HPI skin samples from the input along the first component and the separation of the 5 HPI from the 24 HPI skin samples along the second component (Fig. 2A). These data suggest that the transcriptional profiles between our analyzed timepoints are significantly different from each other.

There were global bacterial changes in transcription at both 5- and 24-hour post-inoculation on mouse skin (Fig. 2; Table S1). At 5-hour post-inoculation, there were 552 upregulated genes and 836 downregulated genes compared to the input (log_2_ fold-change ≦ 1, *P* ≦ 0.05). Using the same cutoff criteria, at 24 hours, there were 578 upregulated genes and 667 downregulated genes compared to the input. When comparing the transcriptomic changes between 5 and 24 hours, there were 220 upregulated genes and 362 downregulated genes using the above thresholds.

*S. aureus* encodes a cyclic peptide-based quorum-sensing system called the accessory gene regulon (*agr*) that activates the expression of extracellular proteases, hemolysins, and other virulence factors (23). Known genes previously demonstrated to be under the control of the Agr quorum-sensing system were significantly upregulated at 5-hour post-inoculation compared to input, and there was no significant difference at 24-hour post-colonization compared to 5 hours (23, 24). Genes encoding virulence factors including the delta-toxin (*hld*) and a multitude of adhesins (*sasA*, *fnbAB*, and *clfA*) were all upregulated at both 5 or 24 hours compared to the input control (Table 1; Fig. 2C and D); all of these genes have been implicated in virulence (25–28). Some virulence genes such as the Panton-Valentine leukocidins *lukF* and *lukS* and the proteases *sspA* and *sspB* were upregulated at 5 hours compared to input, and their expression increased significantly at 24 hours compared to 5 hours (Fig. 2E; Table 1; Table S1).

**TABLE 1:** Select virulence-related RNAseq data

Gene name	Locus tag	5 hours log_2_ fold-change	24 hours log_2_ fold-change	24 hours vs 5 hours log_2_ fold-change	Predicted function
*hld*	SAUSA300_1988	1.16	1.90	0.74	Delta-hemolysin
*sraP* (*sasA*)	SAUSA300_2589	0.75	1.56	0.81	Cell wall anchor domain-containing protein
*sasG*	SAUSA300_2435	3.68	0.36	−3.32	Cell wall surface anchor family protein
*lukS*-PV	SAUSA300_1382	−1.88	2.88	4.76	Panton-Valentine leukocidin
*splD*	SAUSA300_1755	0.78	4.94	4.16	Serine protease SplD
*lukE*	SAUSA300_1769	−1.71	1.27	2.98	Leukotoxin
*lukD*	SAUSA300_1768	0.05	1.35	1.31	Leukotoxin

Upregulation of a variety of genes related to metabolism was noted (Table 2), including members of the urease operon (*ureABCEFGD*) and a multitude of genes related to amino acid anabolism (*ilv* and *leu* genes, *pstS*; Fig. 2B through E). The most highly upregulated genes at both 5 and 24 hours were the members of the *fadXDEBA* locus (Table 2), which Kuiack et al. recently demonstrated enable the metabolism of certain fatty acids, which exist in high concentrations throughout the skin (20).

**TABLE 2:** Select metabolism-related RNAseq data

Gene name	Locus tag	5 hours log_2_ fold-change	24 hours log_2_ fold-change	24 hours vs 5 hours log_2_ fold-change	Predicted function
*fadD*	SAUSA300_0228	9.66	8.03	−1.63	Acyl-CoA synthetase
*fadB*	SAUSA300_0226	11.08	9.73	−1.35	3-Hydroxyacyl-CoA dehydrogenase
*fadA*	SAUSA300_0225	9.50	8.65	−0.85	Acyl-CoA acetyltransferase
*ureA*	SAUSA300_2238	2.86	4.47	1.61	Urease subunit gamma
*argF*	SAUSA300_1062	2.30	3.26	0.96	Ornithine carbamoyltransferase
*fakB1*	SAUSA300_0733	−0.75	−1.45	−0.70	DegV family protein
*gehB*	SAUSA300_0320	1.87	3.30	1.43	Triacylglycerol lipase
*ribE*	SAUSA300_1714	2.01	1.43	−0.58	Riboflavin synthase subunit alpha
*lysC*	SAUSA300_1286	4.02	2.00	−2.02	Aspartate kinase

To assess the host response, we analyzed the murine transcriptomic changes in response to MRSA skin colonization between 5- and 24-hour post-colonization (Table S2). Compared to 5-hour post-colonization, at 24-hour post-colonization, there were 46 host genes upregulated (log_2_ fold-change ≧ 1, *P* ≦ 0.05) and 98 genes downregulated (log_2_ fold-change ≦ −1, *P* ≦ 0.05). PCA of host transcripts did not observe significant separation between the 5- and 24-hour post-inoculation skin samples (Fig. S1A). Supporting the PCA, our RNAseq data revealed a few major transcriptional changes occurring between 5- and 24-hour post-inoculation via volcano plot and gene set enrichment analyses (Fig. S1B through D). Gene set enrichment analysis suggested that upregulated genes at 24-hour post-colonization were enriched in pathways corresponding to heme metabolism and the complement system (Fig. S1D). In contrast, downregulated genes at 24-hour post-colonization were enriched in pathways corresponding to host fatty acid metabolism, adipogenesis, and TNF⍺ signaling (Fig. S1D). The generally mild host response to MRSA during skin colonization highlights the intrinsic strength of the innate skin barrier in preventing invasion and infection by MRSA without the need to mount a broad adaptive immune response.

### RNAseq confirmation by functional promoter-fusion assays

To confirm the observed upregulation of the MRSA *fadXDEBA* locus in our RNAseq data, we functionally tested promoter activity upon exposure of MRSA to the host environment using a transcriptional fusion reporter. Vector pHC125 encodes the entire *Photorhabdus luminescens lux* operon under a hybrid promoter (P_CP25-cap5_) and can be stably integrated into the genome at the bacteriophage ɸ11 attachment site to measure constitutive *lux* expression in *in vivo* experiments (29). Using pHC125 as a template, we removed the constitutive promoter and replaced it with a multiple cloning site, generating vector pTJE. Plasmid pTJE was then used to clone different promoters of interest upstream of the *lux* operon to assess transcriptional activity *in vivo*.

Using this reporter construct, we assessed the expression of luciferase under the putative *fadB* promoter, as it has recently been suggested that *fadBA* may be under different transcriptional control than *fadXD* and *fadE* (20). The log_2_ fold-changes of genes in the *fadXDEBA* locus observed in this RNAseq and a previously published data set suggest differences in the regulation of *fadBA* compared to upstream genes (Fig. S2A) (27). Additionally, in *S. aureus* and related species, there is a ~185 bp highly conserved intergenic region between *fadE* and *fadB*, which is longer than the ~45 bp intergenic space observed between these genes in coagulase-negative staphylococci (Fig. S2B through D). Furthermore, it was shown previously that *fadB* may be regulated independently of the other genes within the *fadXDEBA* locus in a CcpA-dependent manner (30). To assess whether there is a promoter element directly upstream of *fadB* and confirm *fadB* transcription *in vivo*, we cloned the putative promoter region of *fadB* into the pTJE vector (hereafter termed P*_fadB_*). As a control, we also cloned the previously characterized Agr P3 promoter in pTJE (hereafter termed P*_agrP3_*) and utilized this construct as a positive control for expression on murine skin (31).

**Fig 3:**
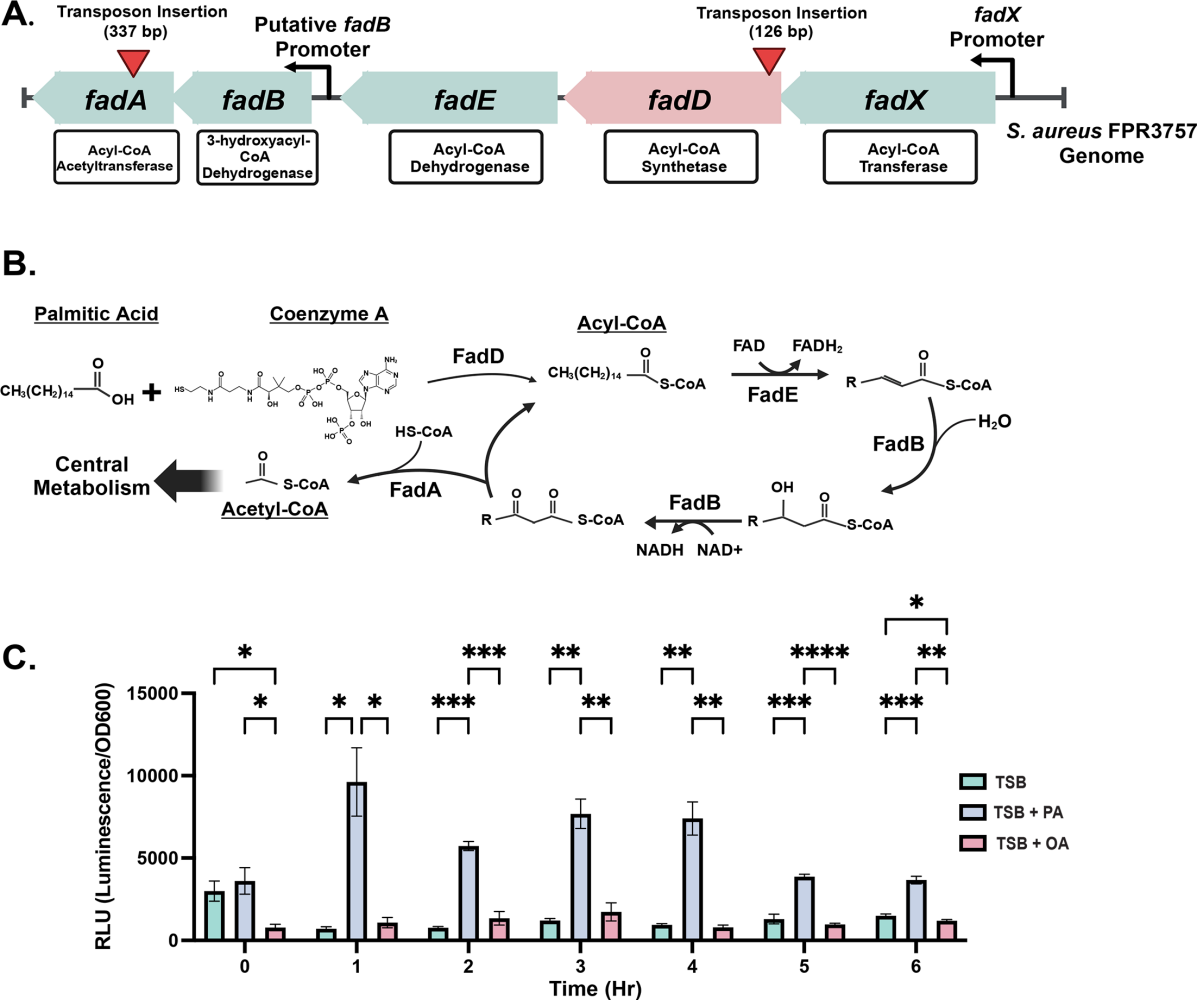
Overview of the *fadXDEBA* locus and β-oxidation. (A) Chromosomal arrangement of the *fadXDEBA* locus from the *S. aureus* FPR3757 reference genome. (B) Biochemical overview of the canonical β-oxidation pathway. (C) Comparison of the P*_fadB_* promoter activity due to incubation with either 500 µM palmitic acid (PA) or 500 µM oleic acid (OA) compared to tryptic soy broth (TSB) + solvent control alone. * Indicates *P* ≤ 0.05, ** indicates *P* ≤ 0.01, *** indicates *P* ≤ 0.001, and **** indicates *P* ≤ 0.0001 by two-way RM ANOVA. Shown are the mean ± SD for three independent biological replicates per condition.

Upon construction and integration of the pTJE promoter-fusion constructs into the MRSA chromosome, we sought to assess *fadB* promoter activity *in vitro*. Previously, the skin-relevant fatty acid, palmitic acid, was shown to upregulate the *fadXDEBA* locus via the promoter upstream of *fadX* (Fig. 3A) (20). As FadB is predicted to function in β-oxidation as a member of the *fadXDEBA* locus (Fig. 3B), we reasoned that the *fadB* promoter would also respond to palmitic acid. In agreement with our hypothesis, we observed a significant increase in luminescence in the P*_fadB_* reporter in response to palmitic acid during growth (Fig. 3C; Fig. S3). There was no change in luminescence in the presence of the unsaturated fatty acid, oleic acid (Fig. 3C). We did observe a slight growth delay of the P*_fadB_* reporter strain grown in both oleic acid and palmitic acid compared to the solvent-only control (Fig. S3). Overall, these data provide supporting evidence for a promoter upstream of *fadB* and also show that the addition of palmitic acid induces P*_fadB_* promoter activity.

**Fig 4:**
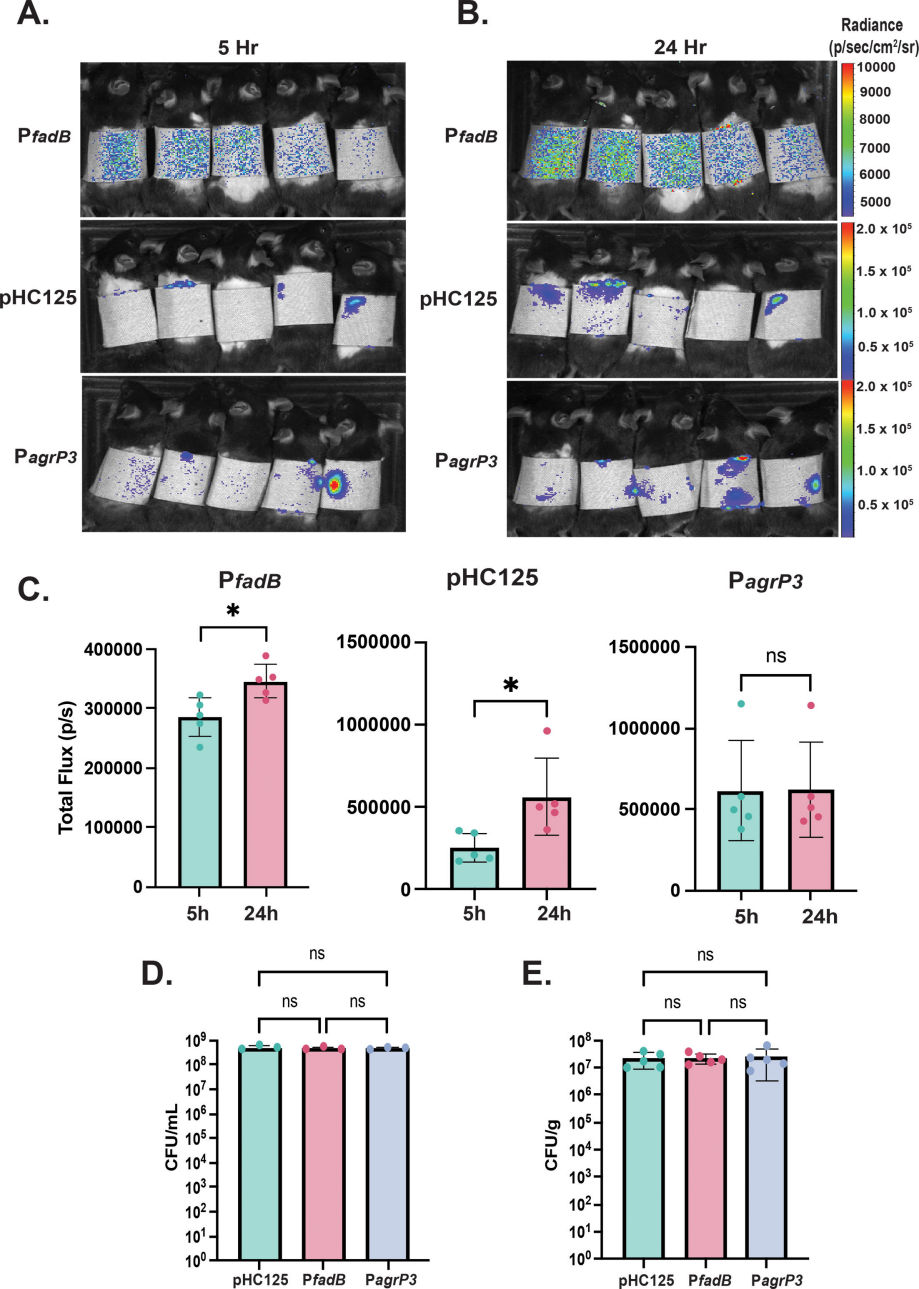
*In vivo* confirmation of key RNAseq hits. (A) Representative photos showing luminescence of healthy C57BL/6J mice colonized epicutaneously with MRSA P*_fadB_* and P*_agrP3_* at 5- and (B) at 24-hour post-inoculation. (C) Quantification of the luminescence for mice colonized with P*_fadB_*, pHC125, and P*_agrP3_* as measured by total flux (p/s). * Indicates *P* ≤ 0.05 by a student’s *t*-test. (D) CFU per milliliter of inoculum shows no difference in the amount of CFUs added to the backs of the mice between the pHC125, P*_fadB_*, or P_agrP3_ groups by an ordinary one-way ANOVA (*P* ≧ 0.05). (E) Ending CFU per gram on the back tissue for mice colonized with P*_fadB_*, P*_agrP3_*, and pHC125 reporter strains shows no statistical difference in ending CFU per gram by an ordinary one-way ANOVA (*P* > 0.05). Shown are the mean ± SD for five individual mice per group.

Next, we sought to utilize these constructs *in vivo* to functionally confirm the observed upregulation of the *fadXDEBA* locus and genes controlled by the Agr quorum-sensing system. To do so, we tested MRSA encoding the P*_fadB_* and P*_agrP3_* reporters in our *in vivo* colonization model and measured luminescence using *in vivo* bioluminescent imaging (IVIS). In accordance with both our RNAseq data and *in vitro* studies, we observed a significant increase in luminescence for the P*_fadB_* reporter strain as measured by total flux between 5- and 24-hour post-inoculation of our bioluminescent reporter strains onto healthy murine skin (Fig. 4A through C). Supporting our RNAseq data, we observed no change in luminescence for the P*_agrP3_* reporter strain at 24-hour post-colonization vs the 5 hours, suggesting that Agr activity is fairly constant during these MRSA colonization timepoints (Fig. 4A through C). We also included a strain containing the integrated pHC125 plasmid (containing the *cap5* and the CP25 promoters in tandem) and observed a similar increase in luminescence between 5- and 24-hour post-inoculation (Fig. 4A through C) (29). The MRSA *cap5* locus was largely upregulated at 24-hour post-inoculation in our RNAseq data set, which supports our observations that the pHC125 luminescence also increased over time upon inoculation onto the skin. Furthermore, numerous capsule biosynthesis genes in *S. aureus* strain MRSA252 have been shown to be highly upregulated in response to other skin fatty acids such as linoleic acid and oleic acid, again supporting our IVIS and RNAseq observations (32). Therefore, our RNAseq data agree with the observed increase in luminescence from pHC125 as well as previous literature (32). The observed changes in luminescence are not due to differences in either the starting CFU per milliliter applied to the skin (Fig. 4D) or the final CFU per gram obtained from harvested skin at 24-hour post-infection (Fig. 4E), suggesting that the differences in luminescence may be due to promoter modulation. Overall, our results using a combination of both *in vitro* and *in vivo* approaches are consistent with the upregulation of the *fad* locus in the RNAseq data set.

### Investigating the role of members of the *fadXDEBA* locus in resistance to oxidative stress

It had previously been reported that *S. aureus* cannot β-oxidize fatty acids as a nutrient source (33, 34). However, recent studies indicate that the skin-relevant fatty acid, palmitic acid, can be degraded by *S. aureus* via conventional β-oxidation (Fig. 3B) (20). Despite this new knowledge, our current understanding of the role of the *fadXDEBA-*mediated β-oxidation on MRSA physiology is limited. Because the *fadXDEBA* loci are the most highly upregulated MRSA genes in our model, we sought to confirm previous data on the *fadXDEBA* locus and expand on how MRSA β-oxidation may affect the cell. We began by testing for a colonization phenotype for *fadB::*Tn compared to MRSA WT in our healthy skin colonization model using IVIS imaging. Although we observed no decrease in *fadB::*Tn bacterial burden by bioluminescence, we observed a slight decrease in skin transepithelial water loss in the mutant, though this phenotype is subtle and only presents early during colonization (Fig. S4). Importantly, this experiment was conducted over the course of 4 days, demonstrating the utility of our skin colonization model for experiments of longer duration.

Next, we sought to take advantage of the critical role of coenzyme A (CoA) in the β-oxidation pathway. It has been previously shown that CoA encompasses a large proportion of the total small molecular weight thiol pool of *S. aureus* (35, 36). We reasoned that targeted disruptions in an induced β-oxidation pathway would alter CoA availability in the cell, altering the MRSA response to intracellular pools of reactive oxygen species. The antibiotic fosfomycin has been shown to increase the intracellular levels of hydroxyl radicals (37), and the thiol pool in *S. aureus* is known to protect MRSA against the harmful effects of oxidants and maintain a reduced intracellular environment (38). Therefore, we hypothesized that targeted disruptions in MRSA β-oxidation may affect tolerance to fosfomycin and other oxidative stressors.

**Fig 5:**
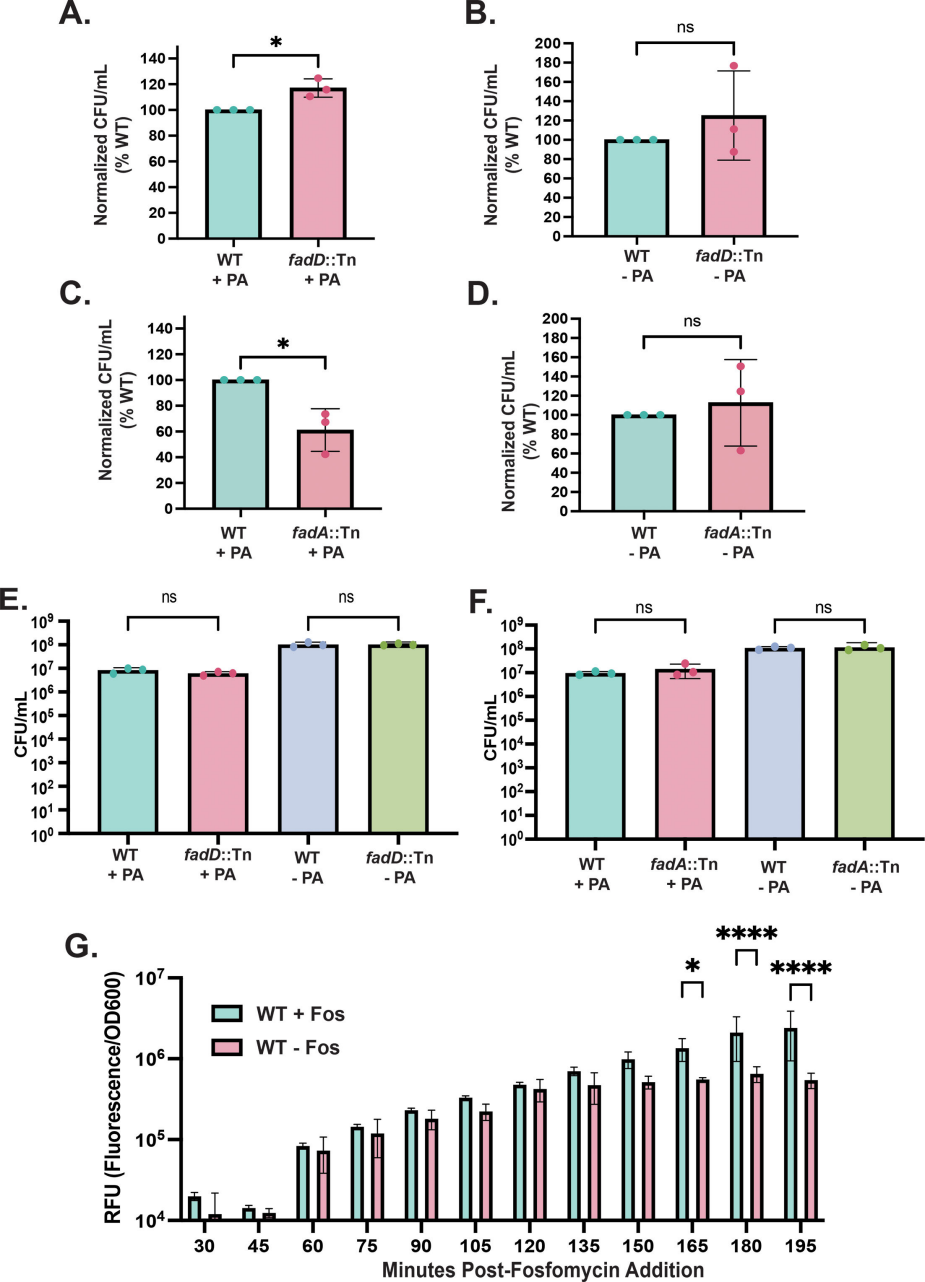
Manipulation of *fadD* and *fadA* modulates fosfomycin resistance. (A) *fadD::*Tn is more resistant to fosfomycin when grown in the presence of 500 µM palmitic acid (PA) compared to WT, but not without (B) when comparing normalized CFU per milliliter for each experiment. (C) *fadA::*Tn is more susceptible to fosfomycin compared to WT when grown in the presence of 500 µM PA but not without (D) when comparing normalized CFU per milliliter for each experiment. (E and F) The differences observed in panels A through D are not due to differences in starting CFU per milliliter for each condition. (G) Incubation of MRSA with fosfomycin increases oxidative stress as indicated by increased fluorescence of 2′,7′-dichlorodihydrofluorescein diacetate. * indicates *P* ≤ 0.05 as measured by a student’s *t*-test for panels A–D. For panels E and F, statistics were performed using an ordinary one-way ANOVA, where * indicates *P* ≤ 0.05. For panel G, * Indicates *P* ≤ 0.05, and **** indicates *P* ≤ 0.0001 by a two-way RM-ANOVA. ns Indicates *P* > 0.05. Shown are mean ± SD for three independent biological replicates per condition.

To test this hypothesis, we utilized MRSA transposon mutants of *fadD* (*fadD::*Tn) and *fadA* (*fadA::*Tn) and assessed sensitivity to fosfomycin when grown in the presence of palmitic acid (Fig. 3A). When induced, FadD catalyzes the first committed step of β-oxidation by ligating a fatty acid to CoA, thus locking a portion of the CoA pool within the β-oxidation pathway (Fig. 3B). Blocking this first step would maintain the high intracellular CoA pool for MRSA resistance to hydroxyl radicals. In agreement with this hypothesis, we observed that *fadD::*Tn has increased relative resistance to fosfomycin treatment compared to WT, and this phenotype is dependent on the bacteria growing in the presence of palmitic acid (Fig. 5A and B). Additionally, this phenotype is not due to differences in starting CFU per milliliter, suggesting that either intracellular CoA is protective against fosfomycin-mediated oxidative stress, or FadD has other roles for protection of MRSA against fosfomycin (Fig. 5E). Furthermore, we utilized a mutant in *fadA::*Tn in the same assay, as FadA catalyzes the final step in β-oxidation, and a mutation would trap CoA in the pathway. Using the *fadA* mutant, we observed a significant decrease in the relative resistance to fosfomycin in a palmitic acid-dependent manner compared to WT (Fig. 5C and D). Again, these results are not due to differences in starting CFU per milliliter (Fig. 5F) but rather suggest that there may be modulation of the protective intracellular CoA pool when MRSA utilizes palmitic acid. Importantly, we were able to detect a significant increase in intracellular oxidative stress caused by fosfomycin using the oxidative stress-sensitive dye, 2′,7′-dichlorodihydrofluorescein diacetate (Fig. 5G), supporting the concept that fosfomycin does increase oxidative stress during antibiotic application.

**Fig 6:**
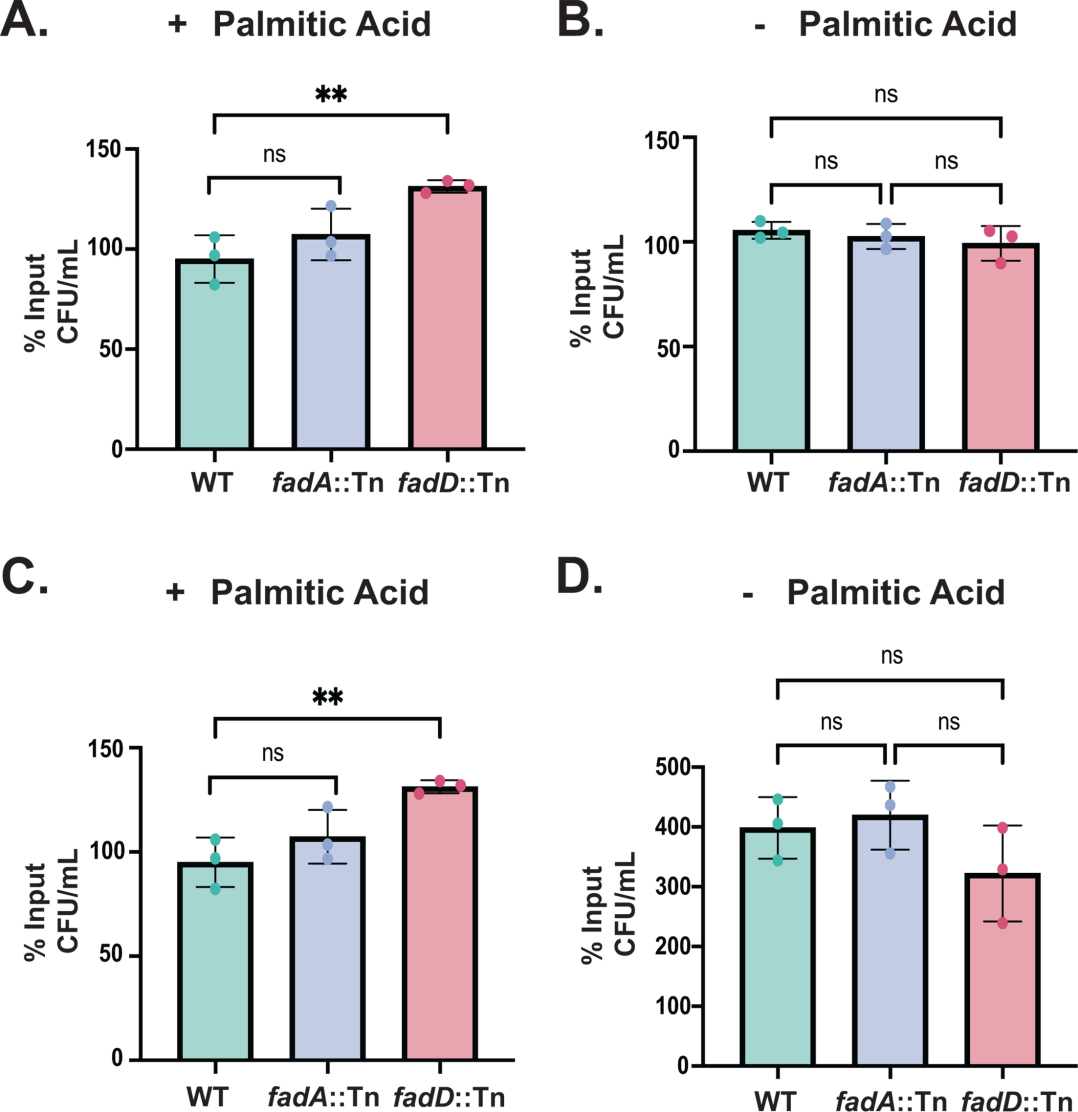
*fadD::*Tn is more susceptible to NaOCl and paraquat stress. (A) *fadD::*Tn is more resistant to killing by NaOCl in the presence of 500 µM palmitic acid; however, in the absence of palmitic acid, each of the strains tested is equally inhibited and is generally more tolerant to NaOCl challenge (B). (C) *fadD::*Tn also displays enhanced survival in the presence of paraquat in a palmitic acid dependent manor, as growth in media lacking palmitic acid renders paraquat not as effective in inhibiting MRSA with no differences in paraquat inhibition observed between strains in media lacking palmitic acid (D). * Indicates *P* ≤ 0.05, and ns indicates *P* > 0.05 as measured by a student’s *t*-test for panels A and C. For panels B and D, statistics were performed using an ordinary one-way ANOVA, where ns indicates *P* > 0.05. Shown are mean ± SD for three independent biological replicates per condition.

Though we observed a fosfomycin-dependent increase in the intracellular oxidative stress, we sought to test whether *fadD::*Tn or *fadA::*Tn are differentially susceptible to either sodium hypochlorite (NaOCl) or N,N′-dimethyl-4,4′-bipyridinium dichloride (paraquat), which are known to directly induce oxidative stress as their primary mechanism of toxicity (39–42). Consistent with our fosfomycin observations, we observed that *fadD::*Tn is more resistant to both NaOCl and paraquat stress when grown in the presence of palmitic acid compared to WT (Fig. 6A and C). However, the *fadA::*Tn showed no significant difference in susceptibility to both NaOCl and paraquat from WT. This may be due to the intrinsic variability of oxidative stress responses in bacteria or may suggest an unknown stress response mechanism that differs between fosfomycin and NaOCl or paraquat in which FadA is important, or the putative function of FadA may be satisfied under NaOCl or paraquat stress by another unknown enzyme in the *fadA::*Tn mutant.

Together, these data may support a previously undescribed role for members of the MRSA *fadXDEBA* locus in resistance to oxidative stress, and skin-relevant fatty acids such as palmitic acid effectively induce the pathway. Our data suggest that bacterial growth in the presence of the skin fatty acid palmitic acid modulates MRSA susceptibility to fosfomycin-mediated killing as well as to killing caused by NaOCl and paraquat. Interestingly, we observed that palmitic acid significantly sensitizes all strains tested to oxidative stress (Fig. 6), suggesting that overall, skin-relevant fatty acids may synergize effectively with oxidative stress agents against *S. aureus* during skin colonization.

## DISCUSSION

*S. aureus*, and its antibiotic-resistant derivative MRSA, are successful nasal and skin colonizers of humans and the most common skin pathogens. The ability to rapidly alter gene expression in response to environmental cues enables this pathogen to survive in these diverse niches. Despite decades of studies on how *S. aureus* causes disease, an understanding of the transcriptional changes that occur during skin colonization remains unknown. In this study, we showed using an unbiased transcriptomics approach that MRSA skin colonization results in global changes to metabolism and virulence, findings that were confirmed using *in vitro* promoter fusion experiments as well as *in vivo* bioluminescent imaging. Our findings revealed that MRSA induces β-oxidation on the skin, and we demonstrate a role for this pathway in managing oxidative stress. Furthermore, we identified a new upstream promoter element ahead of *fadB*, with the *fadB* promoter region being activated by the addition of the saturated fatty acid palmitic acid but deactivated by the addition of the unsaturated fatty acid, oleic acid. Thus, it appears that the regulation of all the members of the *fadXDEBA* locus may be multifaceted and more complex than previously thought.

During periods of transient skin colonization, *S. aureus* shows a remarkable capacity to rapidly modulate its transcriptome in response to new nutrients and environmental conditions, as well as transition to an active infection via the upregulation of crucial virulence factors. O’Gara recently posited that MRSA establishes a “beachhead” during skin colonization to prepare for the translocation into and infection of other body niches (17). We observed that a multitude of known and important virulence factors such as the proteases, toxins, and adhesins are upregulated significantly upon colonization, many of which become highly upregulated between 5- and 24-hour post-inoculation. The rapid response of MRSA to the skin environment is likely due to increased activation of a variety of two-component regulators. This demonstrates that modulatory signals for these two-component systems exist in the skin environment, and the gene products under the regulation of these systems are broadly necessary during skin colonization and possibly infection. For example, *saeP* is upregulated at 24-hour post-inoculation, which may demonstrate increased activation of the *saeRS* two-component system on the skin. Interestingly, the *saeRS* two-component system is known to regulate the production of proteins known to be important for skin infection or adhesion to human corneocytes such as coagulase (*coa*) and fibronectin-binding proteins A and B (*fnbAB*), respectively (27, 43, 44). Importantly, *coa* and *fnbAB* were significantly upregulated at 5-hour post-colonization (Table S1). We observed constant activity of the Agr system upon inoculation onto murine skin (Fig. 4; Table S1), suggesting that Agr-regulated virulence factors are constantly produced during skin colonization. When considering the overarching changes in virulence gene expression during colonization, our RNAseq data suggest that upon inoculation onto the skin, MRSA immediately begins priming for virulence by upregulating a multitude of functionally diverse virulence factors.

Apart from known virulence genes, we also observed global changes to MRSA metabolism, which may help to decipher the metabolic requirements when this pathogen encounters the nutrient-limiting and antimicrobial skin environment. The most highly upregulated genes in our RNAseq data were those within the *fadXDEBA* locus, which are likely to be involved in conventional β-oxidation of certain fatty acids. We showed that, through sensitivity differences to fosfomycin, NaOCl, and paraquat, bacterial growth during palmitic induction may alter MRSA thiol pools, thus translating to differences in resistance to reactive oxygen species. This may be particularly important during the neutrophil response to *S. aureus*, as *S. aureus* resistance to oxidative stress on the skin has been shown to be important for its persistence during skin colonization (45). Of note, it appears that skin fatty acids may synergize with oxidative stressors, with NaOCl and paraquat being relatively more toxic to even WT MRSA in the presence of palmitic acid than without it. It makes intuitive sense that MRSA might utilize fatty acids on the skin to produce energy, as the skin environment has a high concentration of these energy-dense molecules. However, the metabolism of host fatty acids may sensitize MRSA to oxidative stress.

Aside from members within the *fadXDEBA* locus, other metabolic genes were upregulated during the colonization of skin at both 5- and 24-hour post-inoculation. These include genes involved in urea metabolism (*ureB*, for example)*,* siderophore biosynthesis (the *sbn* genes), and genes related to branched-chain amino acid biosynthesis (the *ilv* and *leu* genes). Together, these adaptive changes to the MRSA transcriptome show a shift toward utilization of host-relevant nutrients such as fatty acids and the biosynthesis of key amino acids during bacterial growth. The urease operon is regulated by the *agr* quorum-sensing system and is important as a stress tolerance system in acidic environments (46). The observed upregulation of genes related to the biosynthesis of branched-chain amino acids may indicate that these nutrients are limiting on the skin, and the biosynthetic genes are essential to MRSA during colonization. In contrast, the mouse RNAseq data show that the host is responding to MRSA colonization by upregulating genes involved in nutrient sequestration. The divergent metabolic response of MRSA and the host suggests that the skin barrier and bacteria are in a tight interplay for nutrient scavenging and innate immune evasion despite colonization manifesting as a macroscopically noninflammatory and benign event.

Though our RNAseq data serve as an early roadmap for understanding MRSA colonization, there remains much work in confirming the important genes for colonization, as well as understanding how each of the gene products contributes individually to *S. aureus* colonization and infection. Our model presented in this work can be used to broadly study the interactions between host and pathogen during skin colonization. Though our RNAseq model was conducted over the course of 24 hours, we showed that MRSA can be detected by IVIS *in vivo* bioluminescent imaging on murine skin for at least 4 days (Fig. S2), demonstrating the versatility in our model. The ability to study both long- and short-term colonization by MRSA may be of great utility to those wishing to study not only MRSA and mouse genes of interest but also anti-colonization and practical antibacterial therapeutics against MRSA at the host-pathogen interface. Furthermore, the use of mice to study skin colonization may be more accessible and affordable than other models such as skin explants or tissue culture. Furthermore, using live mice with intact immune systems may provide a more accurate representation of MRSA skin colonization than using skin explants or tissue-culture models.

We utilized this model of healthy skin colonization to study the MRSA response during colonization; however, this could be used to study other important aspects of skin colonization, such as those related to the immune system and other innate antimicrobial defenses of the skin. Although our murine model for skin colonization can provide insights into how MRSA, and indeed other bacteria, might colonize the skin, our murine model should only be used as a starting point for understanding bacterial skin colonization. Important physiological aspects of the mouse such as the murine immune system (47) and overall skin structure (48) are indeed different than that of humans, which may warrant investigation in other models in tandem with our murine epicutaneous skin colonization model. Therefore, we hope that our epicutaneous model for MRSA skin colonization and RNAseq data set presented here serve as roadmaps to begin to better understand how MRSA colonizes the skin of not only mice but also other organisms.

Though we attempted to show that MRSA β-oxidation may affect the broader bacterial physiology and resistance to antibiotics, this hypothesis requires additional mechanistic study. Furthermore, the extent and use of fatty acid metabolism by *S. aureus* remain unclear, as there are many similarly structured fatty acids commonly found in the skin. Despite these open questions, this work remains critical in understanding the transcriptomic changes in MRSA during colonization and may reveal further targets of therapeutic interest, as well as increase our understanding of how *S. aureus* responds to its introduction to host-relevant niches.

## MATERIALS AND METHODS

### Bacterial strains and general growth conditions

The bacterial strains used in this study are described in Table S3. All overnight cultures were grown shaking at 220 rpm at 37°C under normal atmospheric conditions. All MRSA strains were routinely cultured in 5 mL of tryptic soy broth (TSB, BD ref: 211022) in a sterile 15 mL glass culture tube. When growing *Escherichia coli* for use in plasmid preparation, 25 mL cultures in Lennox L broth (LB, RPI ref: L24066) were grown in 150 mL Erlenmeyer flasks containing the appropriate antibiotic for selection. For strain construction, bacterial transformants and transductants were grown and selected on either LB agar (LBA, RPI ref: L24022, 1.2% agar) for *E. coli* or tryptic soy agar (TSA, BD ref: 236920, 1.5% agar) for MRSA. Media was supplemented with tetracycline at 2 µg/mL or spectinomycin at 50 µg/mL where appropriate.

### Cloning and strain construction

Details on all strains, plasmids, and primers used in this study can be found in Table S3. Cloning of the pTJE plasmids was completed using standard restriction cloning. The pTJE plasmids are based on the pHC125 backbone described previously (29). The *lux* operon in pHC125 is under the partial transcriptional control of the capsule biosynthesis *cap5* promoter, and the *cap* locus is significantly upregulated in our RNAseq data set, so the existing promoters and terminators were removed and replaced with a multiple cloning site, which allows for the testing of native MRSA promoters. To create the pTJE 1 and 2 plasmids, pHC125 was first prepared from AH4800 using the Qiagen Qiaprep Spin Miniprep Kit (Ref: 27104). The *cap5* and *cp25* promoters as well as the endogenous terminator were removed by restriction digestion of 1 µg of pHC125 with PstI-HF (NEB ref: R3140S) and BamHI-HF (NEB ref: R3136S) using the manufacturer-suggested protocol. MRSA promoter sequences were amplified by PCR from genomic DNA (gDNA) extracted from AH1263 with Q5 DNA polymerase (NEB ref: M0491S) using the manufacturer-recommended protocol. P*_agrP3_* was amplified using P*_agrP3_*_F and P*_agrP3_*_R, as have been used previously (31) (Table S3). P*_fadB_* was amplified using P_fadB__F and P*_fadB_*_R (Table S3). PCR products were purified using the Qiagen QIAquick PCR purification kit according to the manufacturer’s protocol. The purified PCR products were digested as above using PstI and BamHI, cleaned using the Qiagen QIAquick PCR purification kit, and the promoters were ligated into pHC125 digested with the same restriction enzymes using T4 DNA ligase (NEB ref: M020S) according to the manufacturer’s protocol. The ligated vectors were transformed into DH5⍺ and plated on LBA as described above. Transformant plasmids were prepared as above, and the promoter insertion was confirmed by whole-plasmid sequencing.

Upon miniprepping the pTJE1 and two plasmids from DH5⍺, the plasmid was electroporated into the *S. aureus* RN4220 strain containing helper plasmid pLL2787 (strain AH2138) (49). Transformants were patched onto TSA plates and grown overnight in TSB as described above. Clones containing the chromosomally integrated vector used as the donor strain in Φ11 phage-mediated transduction of the integrated vector into the *S. aureus* LAC (AH1263) background as previously described (50). All integrates were confirmed by PCR from chromosomal gDNA prepped from the recipient strain using P*_fadB_*_F or P*_agrP3_*_F and pTJE_SeqR (Table S3). The plasmid pTJE1 contains the P*_fadB_* promoter, and the pTJE2 plasmid contains the P*_agrP3_* promoter (Table S3). All transposon mutants used in this study were created via transduction from the Nebraska transposon mutant library (51, 52) using ɸ11 as previously described (50). All transductants were confirmed to have the correct insertion by PCR using purified gDNA and the primers described (Table S3).

### RNAseq model for healthy murine skin colonization by MRSA

Seven-week-old healthy female C57BL/6J mice were obtained from Jackson Laboratories and allowed to acclimate to the housing facility for 1 week. For the RNAseq experiment, five mice were pooled for each of three biological replicates (15 mice total per timepoint). As a control, two saline-only mice were pooled per timepoint.

One day prior to inoculation, mice were anesthetized using isoflurane, and the mouse backs were shaved and depilated with Nair. Removed hair and excess Nair were wiped away prior to returning the mice to cages. On the day of inoculation, three biological replicates of MRSA grown for 16 hours in TSB were subcultured 1:50 (vol/vol) in TSB and grown to an OD of approximately 1.0. The bacteria were pelleted, washed once in 1× PBS, and resuspended in saline to achieve a final inoculation dose of 10^8^ bacteria per mouse. Aliquots of each biological replicate were flash frozen in RNAprotect using dry ice to serve as the input controls.

Mice were anesthetized with isoflurane, and immediately prior to inoculation, a pre-swab of the mice was obtained to assess normal microbial load by soaking a cotton applicator in 500 µL of saline and then rolling the applicator about five times on each side of the depilated mouse back and then plating for CFU per milliliter on TSA and mannitol salt agar (MSA) containing cefoxitin to a final concentration of 5.2 µg/mL. The gauze padding of a sterile Band-Aid was inoculated with either 200 µL of bacteria or saline and was affixed to the shaved mouse with the inoculated gauze making contact with the dorsal skin. This Band-Aid was further secured with a second Band-Aid applied with the gauze contacting the ventral side of the mouse. Mice were returned to their cages for 5 or 24 hours. At each of these timepoints, the 15 inoculated mice and 2 control mice were anesthetized using isoflurane. Band-Aids were removed, the backs were swabbed as above for bacterial enumeration, and the mice were sacrificed humanely. Post-sacrifice, 3 × 6 mm biopsy punches from the mouse backs were obtained, and technical replicates were pooled in RNAprotect. To extract the RNA from the samples, the pooled skin biopsy samples were homogenized, and the RNA was extracted as previously described (53). Samples were sequenced by SeqCenter in Pittsburgh, Pennsylvania, using the same preparative methods as described previously (53).

### Bacterial RNAseq data analysis

Sequencing for the input samples was done on a NextSeq2000 giving 2 × 51 bp reads, 12.5 M paired-end reads, and the 5 hours and 24 hours colonization samples were done with a sequencing depth of 25 M paired-end reads. Demultiplexing, quality control, and adapter trimming were performed with bcl-convert (v3.9.3). The quality of the resulting reads was checked using FastQC (54). Adapter removal and read trimming were performed using Trimmomatic, using the paired-end option with a minimum read length of 36 nucleotides and trimming quality of 3 (55). After trimming, read quality was again assessed with FastQC. Trimmed reads were aligned to the *S. aureus* subsp. *aureus* LAC reference genome (RefSeq: GCF_024391155.1), and read counts were quantified using EDGE-pro (56). Un-normalized read counts were imported into R-Studio, and differential expression between Input, 5 HPI, and 24 HPI samples was quantified using DESeq2 with normalization (57). The PCA plot, heatmap, and volcano plots were generated using ggplot2 (58).

### Host RNAseq data analysis

Transcriptomic results were processed using CLC Genomics Workbench (Qiagen, version 20.0.4). Raw unpaired data were imported, adapter trimmed, aligned, paired, and annotated into CLC Workbench using default settings: mismatch cost, 2; insertion and deletion cost, 3; length and similarity fraction, 0.8. Reads were first mapped to the reference genome *Mus musculus* genome (mm10). Following this step, unmapped reads were mapped to the *S. aureus* reference genome (RefSeq: GCF_024391155.1). Uniquely mapped transcripts were normalized and used for differential gene expression analysis using R (v4.2.2) (59), Rstudio (v2022.12.0 + 353, RRID: SCR_000432), and DESeq2 (v1.36.0, RRID: SCR_015687) (57). Differentially expressed genes were filtered based on an absolute log2 fold change cutoff and Benjamin-Hochberg false discovery rate cutoff. fGSEA (v1.22.0, RRID: SCR_020938) (60) with 10,000 permutations, and the Hallmarks and GO Biological Processes gene set collections from the Molecular Signatures Database were used for mouse pathway analysis (61, 62). Mouse and bacterial sequencing data were deposited to the National Center for Biotechnology Information Gene Expression Omnibus (GEO) with accession number PRJNA1078412. Host RNAseq data were only compared between 5 and 24 hours, as the input-only control groups contained endogenous *S. aureus* upon pre-swabbing. Therefore, we can only make a confident comparison between the 5- and 24-hour groups for the host RNAseq data.

### *In vitro* confirmation of pTJE luminescence and response to palmitic acid

To confirm the bioluminescent activity of the chromosomally integrated reporters, luminescence and growth were assessed *in vitro*. In all experiments using palmitic acid, a 100 mM stock of the fatty acid (Sigma ref: 761195G) was prepared in 70% ethanol. To assess bioluminescence, overnight cultures of the P*_fadB_* reporter strain were diluted 1:100 in 5 mL of sterile TSB buffered with 40 mM 3-(Morpholin-4-yl)propane-1-sulfonic acid (MOPS), pH = 7.0, either containing or lacking palmitic acid or oleic acid (Sigma ref: 75090) to a final concentration of 500 µM or the appropriate solvent. A total of 200 µL of the subcultured bacteria was pipetted into a sterile, black-walled flat bottom 96-well plate (Corning ref: 3603) in three individual technical replicates per biological replicate. The plates were incubated in a Stuart SI505 microtiter plate incubator at 800 rpm and an ambient temperature of 37°C. Luminescence and optical density at 600 nm (OD_600_) were measured every hour in a Tecan Group Ltd. Infinite Pro plate reader. Luminescence was measured using a 1,000 ms integration time with zero attenuation. OD_600_ was measured using a 9 mm bandwidth and 10 flashes. Data are presented as relative luminescent units (RLU, luminescent signal divided by the OD600) for three independent biological replicates for each condition.

### *In vivo* bioluminescent imaging

Seven-week-old healthy female C57BL/6J mice were obtained from Jackson Laboratories and allowed to acclimate to the housing facility for 1 week prior to experimentation. We utilized five mice per condition. One day prior to inoculation, mice were anesthetized using isoflurane, and the mouse backs were shaved and depilated with Nair as above. On the day of experimentation, the pHC125, P*_agrP3_*, and P*_fadB_* reporter strains were subcultured 1:100 into 50 mL of fresh TSB and allowed to incubate at 37°C shaking at 220 rpm until the cultures reached an early-log OD_600_ of approximately 0.1 to avoid preemptively activating the P*_agrP3_* promoter construct prior to inoculation. The bacterial cultures were pelleted at 4°C, washed once with PBS, and adjusted to a final CFU per milliliter of approximately 10^9^ in PBS. The shaved C57BL/6J mice were anesthetized with isoflurane. A total of 100 µL (10^8^ total CFUs) of each bacterial suspension was applied to the sterile pad of a Band-Aid, which was then applied to the shaved back skin of the healthy mice. The inoculated mice were then imaged for bioluminescence using a Xenogen IVIS-200 system with auto exposure, medium binning, and an *f*/stop of 1 in an XIC-3 isolation box under anesthesia. The bioluminescent signal produced by the bacteria on the murine skin was analyzed using the Perkin-Elmer Living Image software version 4.7.4. The total flux (p/s) was calculated from the entire surface of the Band-Aid pad. At 24 hours, the mice were humanely euthanized using CO_2_, at which time the entire shaved back surfaces were removed and placed into 500 µL of sterile PBS contained within a pre-weighed 2 mL bead-beater tube with 1 mm zirconia-silicate beads. The skin samples were weighed and then homogenized three times for 1 minute each, with 5 minutes of rest on ice in between. A total of 20 µL of the resulting homogenate was then serially diluted and plated on mannitol salt agar (BD ref: 211407, 1.5% agar) containing cefoxitin to a final concentration of 5.2 µg/mL to select for MRSA. After approximately 18 hours of inverted static growth at 37°C, the resulting CFUs were enumerated, and these data were used to calculate CFU per gram of back tissue.

### *fadXDEBA* modulation of fosfomycin susceptibility

To determine if MRSA growth in the presence of fatty acids modulates resistance to fosfomycin, we adapted our experiments from a previous protocol (20). In brief, MRSA strains of interest were subcultured to an OD_600_ of 0.01 in 25 mL of TSB containing 500 µM palmitic acid and 0.1% dimethyl sulfoxide (DMSO), or 0.1% DMSO and an equal volume of 70% ethanol as the solvent control for palmitic acid. A total of 500 µM palmitic acid was used, as previous data showed maximum expression of the *fadX* with 500 µM palmitic acid (20). The subcultured bacteria were allowed to incubate at 37°C shaking at 220 rpm for 3 hours. At 3 hours, a sample of pre-treated culture was obtained and serially diluted in PBS for CFU/mL enumeration as the input. Fosfomycin was then added to the cultures at a final concentration of 400 µg/mL. The bacterial cultures were allowed to incubate for 3 hours, after which the cultures were serially diluted in PBS and enumerated for the final post-treatment CFU per milliliter present in the cultures. All stocks of fosfomycin (Sigma ref: P5396) were made fresh for each experiment in Milli-Q water to a concentration of 200 mg/mL and were 0.22 mm filter sterilized prior to use. Data are displayed as normalized CFU per milliliter compared to wild type with normalization calculated per experiment and biological replicate. Data shown are gathered from three independent biological replicates for each strain and condition.

To measure the oxidative stress caused by fosfomycin, the oxidative-stress reactive dye 2′,7′-dichlorodihydrofluorescein diacetate (Sigma ref: D6883) was used as described previously (37, 63). This dye is colorless; however, reaction with reactive oxygen species renders the dye fluorescent in a way that can be measured fluorometrically. To measure fosfomycin production of increased redox stress, 10 µL from an overnight culture of each bacterial strain was added to 5 mL of TSB containing 500 µM palmitic acid and 0.1% DMSO, or 0.1% DMSO and an equal volume of 70% ethanol as above in a sterile 20 × 150 mm borosilicate glass culture tube. The subcultures were allowed to incubate at 37°C shaking at 220 rpm for 3 hours, at which time 5 µM 2′,7′-dichlorodihydrofluorescein diacetate was added from a 5 mM stock in DMSO along with 400 µg/mL fosfomycin prepared as above or no antibiotic as the control. The culture was immediately mixed, and 200 µL/well was transferred to a sterile, black clear-bottomed 96-well plate (Corning ref: 3603). The plates were incubated in a Stuart SI505 microtiter plate incubator at 800 rpm. Every 15 minutes, the OD_600_ and green fluorescence signal of the reacted 2′,7′-dichlorodihydrofluorescein diacetate dye were measured using an excitation wavelength of 490 nm, an emission wavelength of 520 nm with 10 flashes, a gain of 80, and an integration time of 50 μs on a Tecan Group Ltd. Infinite Pro plate reader. Data are presented as RFU (fluorescence signal over the OD600) for three independent biological replicates of each strain and condition.

### *fadXDEBA* modulation of NaOCl and paraquat susceptibility

To assess the role of members of the *fadXDEBA* in resistance to oxidative stress, 10 µL of an overnight culture of the bacterial strains of interest was added to 5 mL of TSB lacking glucose (BD ref: 286220) containing either 500 µM palmitic acid or an equal volume of the solvent only, prepared as above. This media was used as it is more relevant to the glucose-limiting skin environment (27), and glucose has been shown to catabolite repress the *fadXDEBA* locus (20). The bacterial subcultures were allowed to incubate for 3 hours at 37°C shaking at 220 rpm, at which point CFU per milliliter was obtained by serially diluting a sample of each culture and drip plating on TSA, as described above. Then, germicidal bleach containing 8.25% NaOCl (Clorox ref: 30966) was added to a final concentration of 4.4 mM, and paraquat dichloride tetrahydrate (Agilent ref: PST-740) from a 250 mM stock in water was added to a final concentration of 2.5 mM. The cultures were re-incubated at 37°C shaking at 220 rpm for 1 hour, at which point the CFU per milliliter of each culture was measured again. The data shown in Fig. 6 are expressed as a percentage of the input CFU per milliliter for three independent biological replicates of each strain and condition.

## SUPPLEMENTAL MATERIAL

Supplemental figures iai.00028-25-s0001.pdf Fig. S1 to S4.

Table S1 iai.00028-25-s0002.xlsx MRSA RNAseq data.

Table S2 iai.00028-25-s0003.xlsx Host RNAseq data compared between 5 and 24 hours postcolonization.

Table S3 iai.00028-25-s0004.docx Strain, plasmid, and primer information used for this study.

ASM does not own the copyrights to Supplemental Material that may be linked to, or accessed through, an article. The authors have granted ASM a non-exclusive, world-wide license to publish the Supplemental Material files. Please contact the corresponding author directly for reuse.
