# Supplementary material for: Global changes in Staphylococcus aureus virulence and metabolism during colonization of healthy skin
Source: Infect Immun. 2025 Mar 21;93(4):e00028-25. doi: 10.1128/iai.00028-25 (PMC11977313; doi:10.1128/iai.00028-25)
Supplement: Supplemental figures — Fig. S1 to S4. [file iai.00028-25-s0001.pdf]

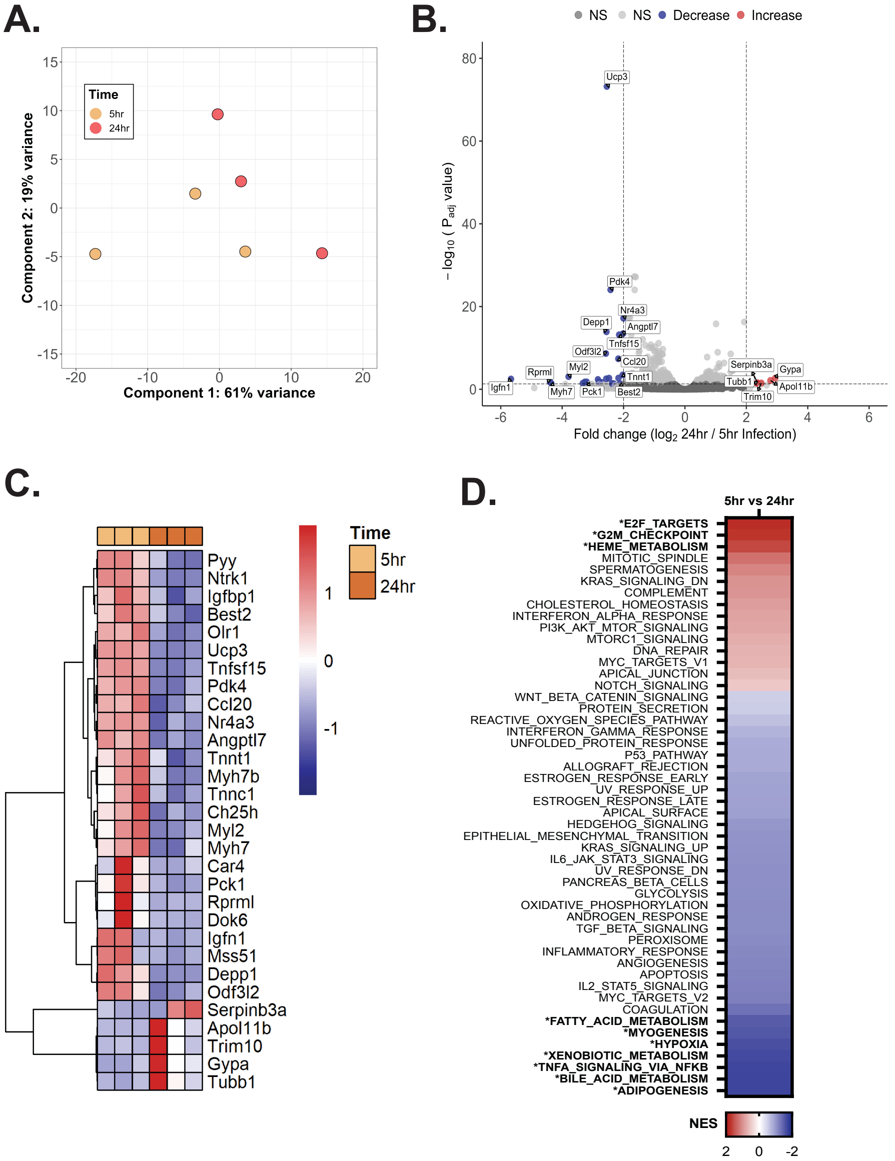

Figure S1: Mouse transcriptional analysis of *S. aureus* skin infections after 5hr or 24hr hours. A) Principal-component analysis of Mouse skin infection. (B) Volcano plot of differentially expressed, (|log2FC| >2 and Adj. p-value <0.05) genes between 5hr and 24hr skin infections. (C) Heatmap of the top differentially expressed genes. Normalized counts are transformed with Z-score method. (D) Gene set enrichment analysis (GSEA) was performed on the fold change of 5hr and 24hr skin infections to calculate the normalized enrichment score (NES).

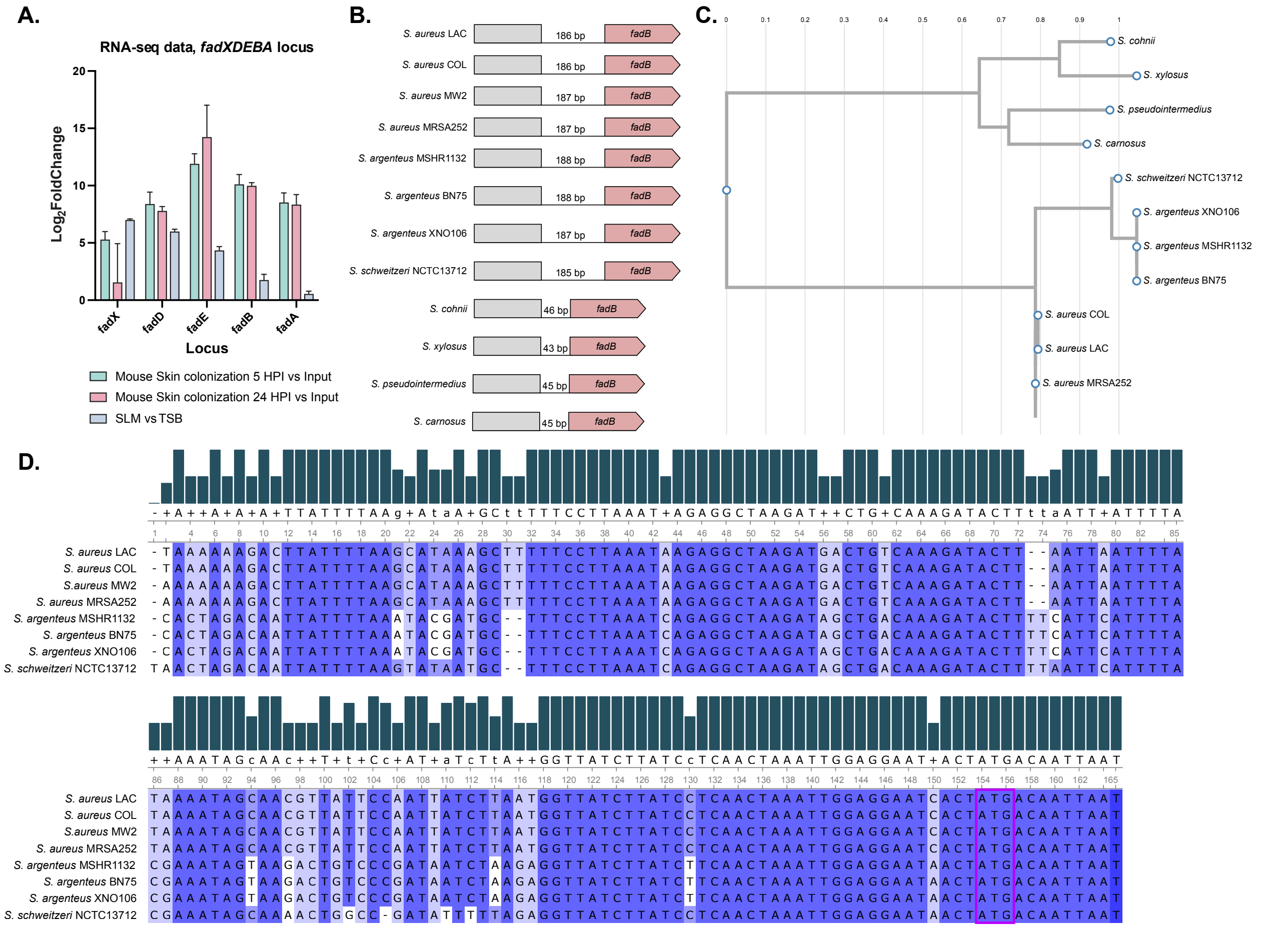

Figure S2: Transcriptional data and overview of the *fadB* upstream DNA region. A) Log2-fold changes of each gene in the *fadXDEBA* locus in the following RNA-seq comparisons: Mouse skin colonization 5 HPI vs. input (this work), mouse skin colonization 24 HPI vs Input (this work), and skin-like media (SLM) vs. TSB (1). All Log_2-fold changes shown were significant in their datasets using a p_{adj} <0.05. B) Repeseentative figures depicting the length of the intergenic region upstream of the *fadB* locus in four *S. aureus* strains as well as strains from other staphylococcal strains. C) A maximum-likelihood phylogeny tree analyzing the 150 bp of the region upstream of the *fadB* start codon for each strain. D) An alignment of the eight long intergenic regions included in the analysis. The red box outlines the *fadB* start codon.

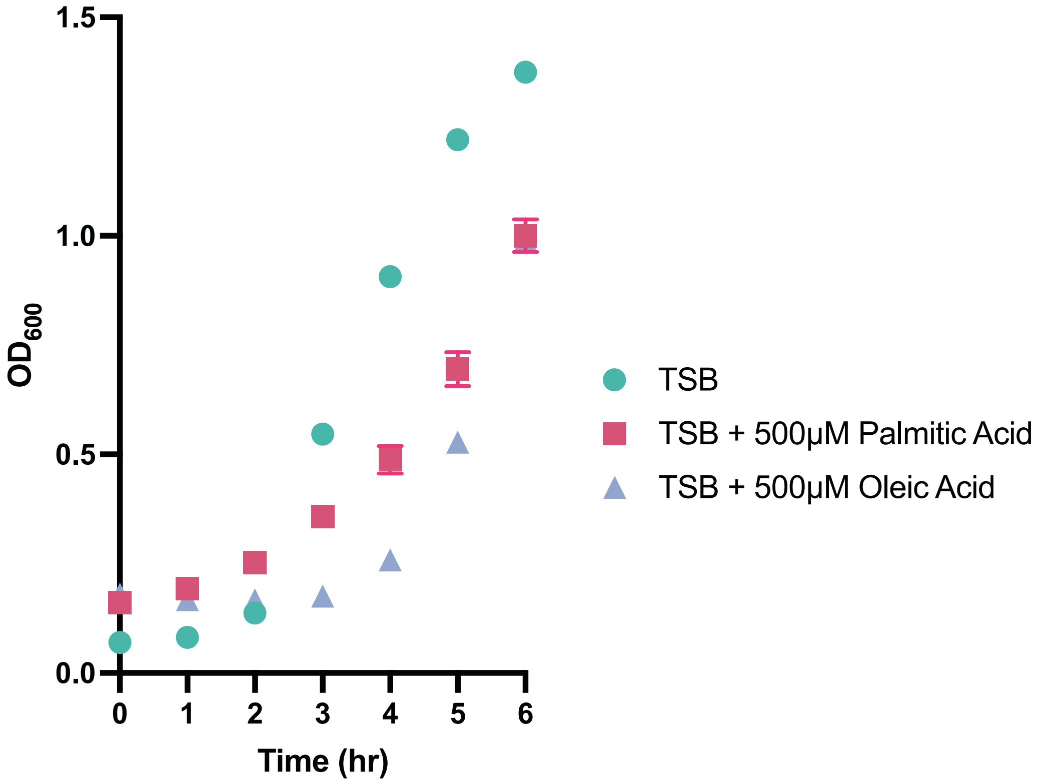

Figure S3: Growth Curves of Reporter Strains. Growth of P_{fadB}, in TSB either containing 500 µM palmitic acid, 500 µM oleic acid, or the TSB + solvent-only control.

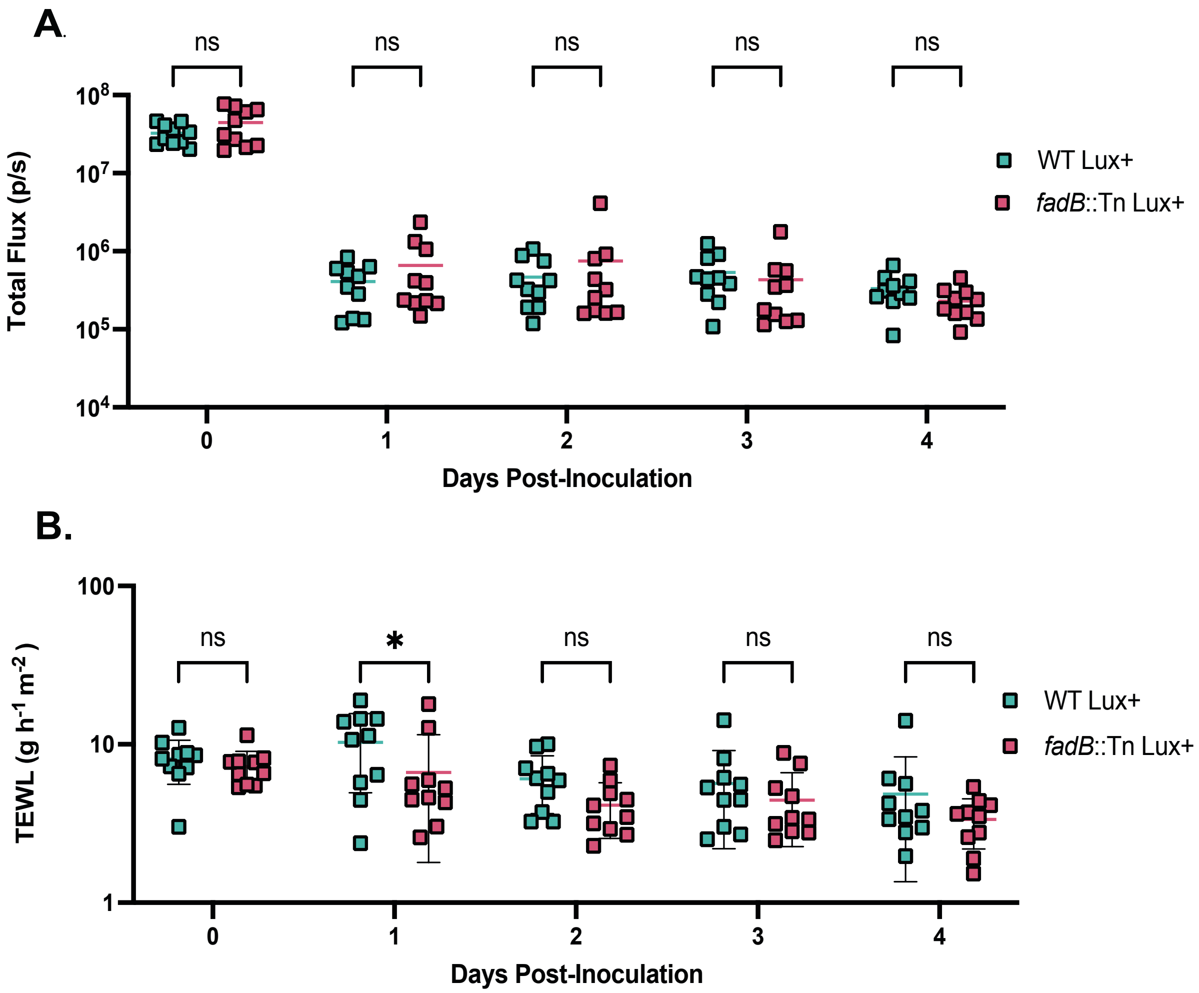

Figure S4: Murine healthy skin colonization model for *fad* gene mutants. A) IVIS bioluminescence as measured by total flux (photons/second) for healthy C57BL/6 mice inoculated with equal CFU's of either WT Lux+ or *fadB*::Tn Lux+ over a time course of four days. B) Transepithelial water loss (TEWL) measured as a proxy for barrier disruption from the back skin of the same mice as in panel A. Shown are the mean ±SD for 10 individual mice, * indicates $p \leqq 0.05$ as measured by a repeated measures two way ANOVA, which was the statistical test used for both panels in this figure.

Supplemental Figure References

1. Costa Flavia G, Mills Krista B, Crosby Heidi A, Horswill Alexander R. 2024. The Staphylococcus aureus regulatory program in a human skin-like environment. mBio 15:e00453-24.
