# Supplementary material for: Global changes in Staphylococcus aureus virulence and metabolism during colonization of healthy skin
Source: Infect Immun. 2025 Mar 21;93(4):e00028-25. doi: 10.1128/iai.00028-25 (PMC11977313; doi:10.1128/iai.00028-25)
Supplement: Table S3 — Strain, plasmid, and primer information used for this study. [file iai.00028-25-s0004.docx]

| **Strain, plasmid, and primer names** | **Description/sequence (5’->3’, restriction sites underlined)** | **Reference/Source** |
| --- | --- | --- |
| *Escherichia coli* strains  DH5⍺  AH4800 | *recA*-deficient cloning strain  DH5⍺ containing pHC125 | New England Biolabs  [1] |
| *Staphylococcus aureus strains*  AH1263  AH6488  AH6489  AH6487  AH6486  AH4807  NE539  NE1092 | WT MRSA, Plasmid-cured derivative of MRSA LAC  *fadA* (SAUSA300_0225)*::*Tn  *fadD* (SAUSA300_0228)*::*Tn  LL29::pTJE1  LL29::pTJE2  LL29::pHC125  JE2 SAUSA300_0228::Tn  JE2 SAUSA300_0225::Tn | [2]  This study  This study  This study  This study  [1]  [3, 4]  [3, 4] |
| Plasmids  pTJE1  pTJE2 | pTJE::P*_fadB_* Reporter  pTJE:: P*_agrP3_*Reporter | This study  This study |
| Primers  P*_agrP3_*_F  P*_agrP3_*_R  P*_fadB_*_F  P*_fadB_*_R  pTJE_SeqR  *fadD_TnCheck_F*  *fadD_TnCheck_R*  *fadA_TnCheck_F*  *fadA_TnCheck_R* | cctctatgctgcagctgtcattatacgatttagtacaatc  Tggcggttggatccttaaacaactcatcaactattttcc  taaacacgctgcagatagcaaataattatatgagatgcattaatttc  agcgggatggatccgttgaggataagataaccattaag  gcgagaaccaagttcattttc  aattcctcctaaaaataatatga  gtatgaaagtgaagctaaag  aaattgcttcaacccgcttc  aaaactggtaagccattacg | [5]  [5]  This study  This study  This study  This study  This study  This study  This study |

**Table S3: List of strains, plasmids, and primers used for this study.** Included are information about restriction sites engineered into the primers (underlined).

**Supplemental Table S3 References**

1. Miller, R.J., et al., *Development of a Staphylococcus aureus reporter strain with click beetle red luciferase for enhanced in vivo imaging of experimental bacteremia and mixed infections.* Sci Rep, 2019. **9**(1): p. 16663.

2. Boles, B.R., et al., *Identification of Genes Involved in Polysaccharide-Independent Staphylococcus aureus Biofilm Formation.* PLOS ONE, 2010. **5**(4): p. e10146.

3. Bae, T., et al., *Generating a collection of insertion mutations in the Staphylococcus aureus genome using bursa aurealis.* Methods Mol Biol, 2008. **416**: p. 103-16.

4. Fey, P.D., et al., *A genetic resource for rapid and comprehensive phenotype screening of nonessential Staphylococcus aureus genes.* mBio, 2013. **4**(1): p. e00537-12.

5. Malone, C.L., et al., *Fluorescent reporters for Staphylococcus aureus.* J Microbiol Methods, 2009. **77**(3): p. 251-60.
